# Influence of early regulatory problems in infants on their development at 12 months: a longitudinal study in a high-risk sample

**DOI:** 10.1186/1753-2000-7-35

**Published:** 2013-10-12

**Authors:** Anna Sidor, Cristina Fischer, Andreas Eickhorst, Manfred Cierpka

**Affiliations:** 1 University Clinic Heidelberg, Institute for Psychosomatic Cooperation Research and Family Therapy, Bergheimerstr. 54, 69115 Heidelberg, Germany

**Keywords:** Early regulatory problems, Early child development, Mother-child-interaction, Maternal distress, At risk

## Abstract

**Background:**

This study examined the extent to which regulatory problems in infants at 4 and 6 months influence childhood development at 12 months. The second aim of the study was to examine the influence maternal distress has on 4-month-old children’s subsequent development as well as gender differences with regard to regulatory problems and development.

**Methods:**

153 mother-child dyads enrolled in the family support research project “Nobody slips through the net” constituted the comparison group. These families faced psychosocial risks (e.g. poverty, excessive demands on the mother, and mental health disorders of the mother, measured with the risk screening instrument *Heidelberger Belastungsskala* - HBS) and maternal stress, determined with the Parental Stress Index (PSI-SF). The children’s developmental levels and possible early regulatory problems were evaluated by means of the Ages and Stages Questionnaires (ASQ) and a German questionnaire assessing problems of excessive crying along with sleeping and feeding difficulties (SFS).

**Results:**

A statistically significant but only low, inverse association between excessive crying, whining and sleep problems at 4 and 6 months and the social development of one-year-olds (accounting for 5% and 8% of the variance respectively) was found. Feeding problems had no effect on development. Although regulatory problems in infants were accompanied by increased maternal stress level, these did not serve as a predictor of the child’s social development at 12 months. One-year-old girls reached a higher level of development in social and fine motor skills. No gender differences were found with regard to regulatory problems, nor any moderating effect of gender on the relation between regulatory problems and level of development.

**Conclusions:**

Our results reinforce existing knowledge pertaining to the transactional association between regulatory problems in infants, maternal distress and dysfunctionality of mother-child interactions. They also provide evidence of a slight but distinct negative influence of crying and sleeping problems on children’s subsequent social development. Easily accessible support services provided by family health visitors (particularly to the so-called “at-risk families”) are strongly recommended to help prevent the broadening of children’s early regulatory problems into other areas of behavior.

## Background

Early regulatory problems are understood as difficulties infants have in adjusting to the environment, regulating their behavior and arousal and in self-calming. These difficulties reveal themselves in symptoms characteristic of age and developmental stages such as crying or sleeping and feeding problems [1]. Crying in the first three months is regarded as the expression of the usual difficulty experienced in initial adjustment to childhood development [2]. However, according to the guidelines of the German Association for Child and Youth Psychiatry [3], *excessive crying/whining* beyond the first 3 to 4 months of life is seen as a regulatory problem in early infancy pertaining to interaction and regulatory contexts such as self-calming, sleeping and feeding. In such a case, the infant would fuss or cry inconsolably and to an excessive degree. The symptoms typically appear two weeks postnatal, peaking in the sixth week, and generally decreasing at the end of the third month [4,5]. As for the prevalence of excessive crying in the first three months, frequencies between 5 and 19% were determined [6]. Persistence of crying beyond the third month was reported in 5.8% of the cases and beyond the sixth month in 2.5% of them [7]. An estimated 5% of all excessive crying cases have organic causes, such as gastrointestinal problems (gastrointestinal reflux, colic), atopy or neuropediatric disorders [5].

Around the third month, most children's self-regulation abilities improve in a surge of development. Excessive crying can be replaced during the course of early childhood development by other symptoms (e.g. sleep disorders) [8]. A study by Kries and colleagues [7] showed that ongoing sleep and feeding problems among children who still cried excessively at 6 months had increased by a factor of 6 to 9.

As with increased crying, the temporary problems relating to the *sleep-wake cycle* represent normal postnatal adjustment difficulties. According to the guidelines for the diagnosis of regulatory disorders, non-organic sleep disorders are only diagnosed from the 6th month since the day-night and sleep-wake cycles are still establishing themselves in the first half of the first year of life [6]. In the second half of the first year (between the 7th and the 9th months), the so-called reorganisation processes set in, which lead to an accumulation of sleep problems involving waking and crying at night [9]. Characteristic problems include falling and/or staying asleep (generally accompanied by crying). Sleeping problems are seen as being related to parental support for falling (and re-falling) asleep: the children are unable to fall asleep on their own. The estimated prevalence of early sleeping disorders in the first two years of life ranges between 10 and 30% [6,10].

*Feeding problems* are also frequently temporary disorders that occur during weaning and introduction of puréed and solid food to the diet. According to the guidelines of the German Association for Child and Youth Psychiatry, a feeding disorder is said to be present when feeding is perceived by the parents as stressful, a meal requires more than 45 minutes and/or the interval between meals is less than 2 hours [3]. The parent–child interaction during feeding is also strained. Due to fear of malnutrition, parents put pressure on the child, contributing to the perpetuation of feeding problems. Since meals in such cases require a great deal of time, the child is fed very frequently, and even during sleep, which results in infants/toddlers lacking hunger as a motivation to eat [6]. Zero to Three [1], a diagnostic system that classifies psychopathological pictures in the first three years of life, distinguished six diagnostic subtypes of feeding disorders, defined by symptoms and clinical course: “feeding disorder of state regulation”, “feeding disorder of caregiver-infant reciprocity”, “infantile anorexia”, “sensory food aversion”, “posttraumatic feeding disorder” and “feeding disorder associated with a concurrent medical condition”.

The prevalence of mild to moderate feeding disorders in the first two years of life is estimated at approx. 15-25% and of serious disorders at 3-10% [11].

### Regulatory problems and parental distress

Excessive crying that continues after the first 3 to 4 months and is often accompanied by sleep-wake-cycle disorders, presents a challenge. It puts a strain on parents and can be a risk factor for the child’s further development [12-14]. In families that are considered to be psychosocially at risk and with access to relatively few resources, early regulation disorders in the children can lead to an escalation and perpetuation of symptoms as well as persistence of regulatory problems in other areas [4]. Von Hofacker and colleagues [3,9] were able to show that the relationship between parents and infant can be seriously influenced by persistent problems coupled with psychosocial pressures. The authors associate “regulatory problems” in early infancy with a triad of symptoms consisting of (1) the influence of the child’s behavior regulation, (2) the occurrence of dysfunctional interaction patterns between the infant and care-giver and (3) parents’ mental and physical stress levels, which are often linked to a current or chronic sleep deprivation. The most significant risk factor comes from interruption of sleep at night owing to irregular childhood sleeping, waking and eating cycles [15-18]. In particular, persistent crying and sleep problems in early infancy affect both the well-being of parents and the relationship between parents and infant [9]. The inability to settle their children and the feelings of helplessness, chronic fatigue, loss of self-confidence and excessive demands cause fears of failure and self-doubt among mothers and fathers with respect to their parental role [19]. Definitions of parental exhaustion vary between extreme fatigue caused by several sleepless nights, which can be remedied by making up for lost sleep, and exhaustion characterised by the fact that it persists even when there is full compensation for the lack of sleep [17]. Since childhood development takes place within the context of a relationship, both difficulties and problems in the relationship as well as the state of the attachment figure can have an effect on development, especially social-emotional development. Salomonsson and Sleed [20] found a strong association between maternal distress and the socio-emotional development of children in the first 16 months of life.

### Influence of regulatory problems on childhood development

Regulatory problems that persist longer than the first 3 to 4 months, present an unfavorable factor for further childhood development. The persistence and “broadening” of the child’s regulatory problems into other areas of behavior contribute to an increased risk for further social-emotional and cognitive development in infancy. With regard to later behavioral problems in children, there are various findings. According to the meta-analysis conducted by Hemmi and colleagues [21], persistent excessive crying has the greatest effect on subsequent symptoms: on externalized problems (*d* = 0.51) and internalized problems (*d* = 0.50) and on ADHD (*d* = 0.42). Feeding problems (*d* = 0.21) and multiple regulatory disorders (*d* = 0.45) were only held in connection with general behavioral problems. Infant sleeping problems in this study had only a small influence on internalized disorders (*d* = 0.24) and general behavioral disorders (*d* = 0.42), while the effect for ADHD was great (*d* = 1.30).

Wurmser and colleagues [8] reported that infants who had received a diagnosis of excessive crying were also judged to be temperamentally “more challenging” at 30 months in comparison to other children. In addition, a greater frequency of both externalizing and internalizing disorders were found in mid-childhood among children who had cried excessively as babies. Desantis and colleagues [22] found an association between duration of whining and unease in the first weeks of life, emotional reactivity and externalizing disorders from the ages of 3 to 8.

In a study by Schmid and colleagues [13], persistent multiple regulatory disorders (increased crying, sleeping and feeding problems in the 5th month) were a predictor of adjustment difficulties and a negative predictor of social skills for pre-school children (56 months). However, this association applied only to boys. The results of the Mannheim Child Risk Study [23] point to an overall more favorable prognosis for an isolated regulatory problem: the behavioral problems rate in later childhood was only slightly higher than among children from the control group. Children with multiple regulatory disorders showed significantly higher rates of subsequent disorders, both internalizing and externalizing. These multiple regulatory disorders nevertheless played a minor role in comparison to the psychosocial pressures on the families involved in the study: the highest rate of mental abnormalities was found among children who had suffered multiple regulatory disorders as infants and who were also subject to high psychosocial risks.

With regard to the long-term effects of early regulatory problems on cognitive development, there is only limited evidence to date and the studies that have been carried out thus far have shown only small or very small effects. Rao and colleagues [12] found a comparatively low cognitive performance (IQ recorded with WPPSI-R) in areas of verbal communication and interaction among five-year-old children with a history of prolonged excessive crying as babies. These children also scored less on fine motor skill development in comparison to other children of the same age. Increased crying only in the first 12 weeks, on the other hand, had no effect on cognitive development.

Wolke and colleagues [14] reported a lower level of development among 20-month-old infants who at the age of 5 months had been diagnosed with multiple regulatory disorders, in comparison to other infants. This association was more pronounced among boys (small effect) than among girls (very small effect) but was significant for both genders. Among 56-month-old girls, a direct inverse association was found between early regulatory problems and cognitive development. In boys, multiple regulatory problems predicted lower mental development at 20 months. The negative influence of early regulatory problems on cognitive development was nevertheless very small. In another study [22], an association was found between duration of whining and unease during the first 12 weeks and sensory perception/stimulus processing at 3 to 8 years old, but no effects of excessive crying were observed.

The etiological mechanisms involved in the long-term effects of early regulatory problems on subsequent emotional and cognitive development in children remain unclear. Excessive crying beyond 3 months is regarded as an indicator of dysfunctional regulatory capacities and potentially low behavioral inhibition, and as an overall predictor of subsequent behavioral abnormalities. It is suspected that ineffective regulatory mechanisms, stimulus hypersensitivity and deficits in behavior regulation play distinct roles in the formation of regulatory disorders (see overview in [21]).

The present study involved children raised in high-risk families, and thus more vulnerable to further stressors and maladaptive outcomes (i.e. [24]). Laucht and colleagues (2004) found the highest rate of mental problems among children who had suffered multiple regulatory disorders as infants; they were also found to be susceptible to high psychosocial risks [23].

### Study aims and hypothesis

This study investigates how and to what extent regulatory problems in 4- and 6-month-old infants affect the children's development at 12 months. Given the limited evidence in the literature, we hypothesize a week association. A differential influence on various aspects of infant’s development, such as motor skills, problem-solving skills and social development is also investigated. On the basis of previous findings, regulatory disorders in the first six months are expected to be associated with a lower level of childhood development at one year. Compared to previous studies [12,14], which used only one measure of cognitive development or general development, the strength of this study is that it seeks to investigate different facets of infant development in the context of regulatory disorders. Due to the paucity of evidence in the literature, the differential influence among the developmental scales is investigated only exploratively.

In addition, given the slight gender-based differences in the link between regulatory disorders and developmental levels [14], we expect to observe a more pronounced association in boys.

Based on other findings [20], we expect, also, to see a link between maternal distress during the children’s 4th month and their subsequent social development at 12 months.

On the basis of the concept of “triad of symptoms” [3,9], we anticipate an association between regulatory problems in infants, maternal distress and dysfunctionality of mother-child interaction.

As the children involved in our study are raised in high-risk families, we seek to investigate to what extent the occurrence of psychosocial risks, such as poverty or low maternal education levels, have an additional effect on the child`s development. If any evidence of a negative impact of early regulatory problems on a child’s development could be found around the infant's first birthday, it would emphasize the importance of early preventive measures in the first year of the child`s life, particularly for those in high-risk families.

The present study builds uniquely upon previous research by examining different facets of infant development in the context of regulatory disorders in a group of younger children raised in high-risk families up to the age of 12 months.

## Methods

### Participants

153 mother-child dyads enrolled in the family support research project “Nobody slips through the net”^a^ made up the comparison group. The participants represented families that were exposed to psychosocial risks such as poverty (income less than 1000€ per household, 35% of the sample), lack of social/family support (27.8%), excessive demands on the mother (50%), mental health disorders of the mother (31.2%) or under-age mothers (6.2%). 52.9% of the families were exposed to only one risk factor, 28.7% to two, 15.4% to three and 2.9% to four risk factors.

### Study design

The data were collected at three intervals: first (T1) at the beginning of the intervention (“Nobody slips through the net” project; see Procedure), when the children were on average 19.04 weeks old (corrected due to prematurity 18.73, *SD* = 2.66), then (T2) at 6.4 months, (*SD* = 0.61) and finally (T3) at 12.39 months (*SD* = 0.67). The dropout rate from the first to the third time of measurement was 13.3% for the comparison group. In terms of socio-demographic variables, the dropout group did not differ from the remaining study sample, indicating that the dropout was not selective.

The study was approved by the Ethics Committee of the Heidelberg University Hospital. Participation in the study was voluntary, and the participants received a small incentive.

The characteristics of the sample are described in Tables 1 and 2.

**Table 1:** Sociodemographic data on the sample (mothers) at the first measurement point (N = 153)

**Age of mothers**	***M (SD)***
	28.2 (6.4)
**Marital status**	***n (%)***
Married	50 (39.1%)
Single mother	29 (22.7%)
Single, partnership with the child’s father	44 (34.4%)
Single, a new partner	5 (3.9%)
**Education**	
Without obtaining qualifications	13 (10.4%)
Secondary general school	47 (37.6%)
Intermediate secondary school	39 (31.2%)
Technical college entrance qualification	5 (4.0%)
University entrance diploma	13 (10.4%)
University	8 (6.4%)
**Monthly income per household**	
<EUR 1,000	41 (35.0%)
EUR 1,000-1,500	43 (36.8%)
EUR 1,500-2,000	16 (13.7%)
>EUR 2,000	17 (14.5%)
**Nationality**	
German	99 (78.0%)
Turkish	7 (5.5%)
Other	21 (16.6%)

**Table 2:** Data on the children after birth and at the first measurement point T1

	**M (SD)**	N*
**Birth during gestational week**	38.77(2.32)	148
**Weight at birth (g)**	3159.31 (634.08)	152
**Age T1 (weeks, corrected due to prematurity)**	18.73 (2.66)	152
	***n (%)***	N*
**Gender/male**	80 (52.3%)	153
**Premature infant (birth < 37 SSW)**	17 (11.5%)	148
**Low gestational weight (< 2500 g)**	23 (15.1%)	152

*The variance of the *N* values depends on different return rates.

### Measures

The *child’s regulatory problems* were recorded by means of a parent questionnaire on regulatory disorders in early infancy - “Questionnaire on crying, feeding and sleep (SFS)” (Groß et al. [25]). The SFS refers to a “typical week” in everyday family life and can be applied within the first year of the child`s life. The Questionnaire contains 52 items (response mode: “1 = never/seldom” to “4 = always”): 3 to capture Wessel’s “rule of threes”, 24 for crying, whining and sleeping (e.g., cry duration, sleep latency), 13 for feeding (feeding problems, concerns about the child’s weight). The remaining 12 items assess the co-regulation, i.e. calming strategies that parents use when their child cries or when it wakes up at night and cannot go back to sleep. The more difficulties children show in terms of crying, feeding and sleeping, the higher the values are in the SFS.

The assessment criteria of the questionnaire, which was constructed on a theoretical and factor-analytic level, were tested on a sample of 642 infants (both clinical and non-clinical subsamples). The factor analysis resulted in three easily interpreted areas: “crying, whining and sleep problems” (Cronbachs α = 0.89), “feeding problems” (α = 0.82) and “co-regulation” (calming strategies of parents against the child’s crying and sleep problems) (α = 0.81). With regard to validity, the SFS distinguished well between the clinical and non-clinical samples. Links were found to exist between the SFS and both diary entries and clinical diagnoses in the clinical sample (parent-infant consultation hours). Because of our interest in regulation problems rather than strategies parents use when their baby cries, this study did not utilize the co-regulation scale.

The *children’s development stage* was measured with the Ages and Stages Questionnaire (ASQ 2^nd^ Edition; Squires and Bricker, [26]). The ASQ aims to gather a child’s development over the span of the first five years of life (from 2 to 60 months). The ASQ is intended to help reveal (in terms of a screening instrument) development deficits, particularly in families at risk, so that interventions can be initiated as early as possible. The questionnaires are filled out by the parents or other guardians, and each consists of 30 items (response mode: “yes” (10), “sometimes” (5), and “not yet” (0)). The following development areas are measured: communication, gross motor skills, fine motor skills, problem-solving skills, and social development. Furthermore, there is an open general question through which parents can express fundamental concerns. The test-retest reliability is 0.90; the inter-rater reliability between a parent and a professional is 0.89; for parents with a low income, it is still 0.85 and can thus be classified as very good. The ASQ shows good concurrent validity with the Bayley Scales of Infant Development (Bayley 2^nd^ Edition, 1993). Sensitivity ranges from 38% to 90% and specificity from 81% to 91%.

The short form of the German version of the standardized parental questionnaire PSI–SF (“Parental Stress Index Short Form,” Abidin, [27]) was used to measure *maternal stress*. This short form consists of 36 items, for which the answer format ranges on a five-level scale from “strongly agree” to “don’t agree at all.” The questionnaire is divided into three subscales: the “parental distress” scale (α = 0.87), the “dysfunctional parent–child interaction” scale (α = 0.80), and the “difficult child” scale (α = 0.85).

The “difficult child” subscale was excluded from analyses due to extensive missing data.

*The general exposure to risk* of the families was measured with the help of the “Heidelberger Belastungsskala” [Heidelberg Stress Scale] (HBS) (reference withheld, [28]). The HBS was developed for a low-threshold and multi-professional assessment of a family’s stress and resources after the birth of a child. It measures the level of family-functioning in the following five areas: (1) child´s stress: illness, disability, prematurity;

(2) parental stress: minor mothers, excessive demands on the parents, mental illness, substance abuse; (3) family stress: lack of family-support, single parent families, chronic or severe illness of a sibling, age difference between siblings lower than 18 months; (4) social stress: poor or no social support, antisocial environment and (5) material stress: poverty, constricted housing conditions.

The responses can be “yes”, “probably“, “no” or “yes” “no”, with values ranging between 0 (no stress) and 100 (very high stress). The following range allocations were set using the HBS: range 0–20: no load; 21–40: small to moderate load; 41–60: middle load; 61–80: high load; 81–100: extremely high load. The HBS shows an excellent inter-rater reliability within a homogeneous professional group (psychology students) (*ICC* = 0.92). As regards construct validity, significant correlations were found with both maternal sensitivity (CARE Index) (*r* = -0.20; *p* = 0.001) and maternal distress (PSI) (*r* = 0.14, *p* = 0.05). In terms of the predictive validity, the risk of taking the child into care in case of high stress in the HBS was increased by 4.5 times (ibid.)

### Procedure

To recruit high-risk families, we approached institutions that had contact with pregnant women and mothers (with newborn children) burdened by psychosocial risk factors. Organizations such as maternity clinics, welfare offices, pregnancy counseling services, midwifery practices, pediatric centers, family support institutions, counseling centers, etc., in Baden-Württemberg, Rheinland-Pfalz, and Hesse were contacted. Because the KfdN intervention areas were meant primarily for families at risk, the burdened families in the comparison group could not be accommodated there.

Furthermore, families in the control group could not be involved in interventions that could be compared with those done by the family midwives in the project area.

Participation of families was sought through cooperating research partners. If we agreed upon a potential family, we sent the contact details to the study’s staff members.

As soon as the consent to contact a family was received from the cooperating institution, the family was contacted by a student assistant specially trained for the task. The participating mothers were informed about the study and data protection regulations at the first appointment in their personal households. The families had to sign the data protection terms and conditions and the participation consent form. Following this, the stress level was assessed (HBS, see Measurement Instruments) (T0). If all the conditions for participation were met (a sufficiently high stress level—i.e., a HBS-value over 20 and adequate language proficiency), the families were contacted again at the first measurement point (T1: child’s age 4 months) and a set of surveys including the SFS, ASQ and PSI-SF was completed.

When the child reached the age of about 6 months (T2), the participating families were contacted again by telephone and a second measurement point was agreed upon, at which point just two questionnaires were to be filled out, SFS and ASQ. Around the child’s first birthday, the families were contacted for the third measurement point (T3: child’s age 12 months), which was conducted in the same way as the first measurement point. At Time 3, parents completed a set of surveys including the ASQ and PSI.

The varying numbers of test participants within the variables presented are the result of varying response rates.

### Statistical methods

For the prediction of children’s developmental levels at T3, the scales for “crying/sleep” and “feeding problems” at T1 and T2 (separate regression models for both measurement times) were tested as predictors (linear regression, method enter hierarchical) using the samples of 97 families at T1 and of 94 at T2. As a first regression model, regulatory disorders (both SFS scales) were tested alone, separately for each ASQ-scale. In the second model, maternal stress level (only at T1) was tested as a potential predictor. For testing gender as a potential moderating variable, gender alone as well as interaction terms gender x “crying” and gender x “feeding” were included in the regression equation in the second model. Potential control and confounding variables, such as developmental level of the child at T1 and T2, premature birth, household income and maternal education level were included in the second model and fitted in the equation. Bivariate association (Pearson correlation coefficients) between both “parental distress” and “dysfunctional parent–child interaction” at T1 and T3 and ASQ-scales at T3 were calculated as well.

For testing the association between early regulatory problem, maternal stress levels and dysfunctional interaction at T1, bivariate Pearson correlation coefficients between “crying/sleeping” and “feeding problems”, “parental distress” and “dysfunctional parent–child interaction” were computed.

The gender differences relating to crying and feeding problems at T1 and T2 as well as the developmental level at T3 were tested with the Mann–Whitney-U-Test owing to unfulfillment of the normal distribution requirement (Kolmogorov-Smirnov-Test significant, see Table 3). T-tests were also conducted.

**Table 3:** Descriptive statistics on regulation problems (T1 and T2), HBS overall stress (T1), maternal stress levels and dysfunctionality of the mother-child interaction (T1) and on children`s developmental levels (T1, T3)

	**M (SD)**	**Range**	**N**	**K-S-Test**
**SFS C/S T1**	1.56 (0.30)	1.00 - 2.50	153	n.s.
**SFS F T1**	1.23 (0.29)	1.00 – 2.69	153	*p* < 0.000
**SFS C/S T2**	1.54 (0.32)	1.04 - 2.75	140	*p* < 0.002
**SFS F T2**	1.28 (0.32)	1.00 – 2.46	140	*p* < 0.000
**HBS O T1**	49.53 (15.00)	10 - 90	150	*p* < 0.000
**PSI PD T1**	2.28 (0.76)	1.00 – 4.58	127	n.s.
**PSI DI T1**	1.40 (0.43)	1.00 – 3.33	126	*p* < 0.000
**ASQ C T1**	51.40	20 - 60	153	*p* < 0.000
**ASQ GM T1**	55.98	20 - 60	153	*p* < 0.000
**ASQ FM T1**	47.58	15 - 60	153	*p* < 0.000
**ASQ PS T1**	53.31	15 - 60	151	*p* < 0.000
**ASQ SD T1**	50.49	15 - 60	152	*p* < 0.000
**ASQ C T3**	44.84 (10.63)	20 - 60	123	*p* < 0.004
**ASQ GM T3**	46.84 (15.78)	5 - 60	122	*p* < 0.000
**ASQ FM T3**	49.71 (10.49)	10 - 60	122	*p* < 0.000
**ASQ PS T3**	47.17 (10.53)	15 - 60	120	*p* < 0.003
**ASQ SD T3**	43.90 (13.44)	10 - 60	123	*p* < 0.006

K-S-Test: Kolmogorov-Smirnov test of normal distribution; SFS: Questionnaires on Crying, Feeding and Sleep; C/S: “Crying / Sleep”; F: “Feeding”; HBS: Heidelberger Belastungsskala; O: Overall stress; PSI: Parental Stress Index; PD : “Parental distress“; DI: “Dysfunctional parent–child interaction”; ASQ: Ages and Stages Questionnaire¸ C : “Communication”; GM: “Gross motor skills”; FM: “Fine motor skills”; PS: “Problem-solving skills”; SD: “Social development”; n.s.: not significant.

For all calculations, a significance level of 0.05 was determined (two-tailed). The statistical analysis of the data was conducted using the statistics program SPSS for Windows, Version 19.0.

## Results

### Descriptive statistics

Table 3 shows descriptive statistics for all variables applied.

Children’s developmental levels at T1 and T3 (see Table 3) were generally similar to the means in the normative ASQ sample [26]. The following percentages of children were classified under the critical cutoff values [ibid.]: communication: 3.3% at T1, 0% at T3; gross motor skills: 6.5% at T1, 5.7% at T3; fine motor skills: 3.9% at T1, 1.6% at T3; problem-solving skills 6% at T1, 5% at T3; social development: 4.6% at T1, 7.3% at T3.

### Relationships between regulatory disorders, maternal stress levels and degree of dysfunctionality of mother-child interaction at T1

Table 4 gives an overview of correlative relationships between regulatory disorders, maternal distress and dysfunctionality of mother-child interaction at the initial measurement T1, T2 and T3. The SFS scale for “crying, whining and sleep problems” correlated with both PSI scales (for “parental distress”, *r* = 0.35, *p* = 0.000; for “dysfunctional parent–child interaction”, *r* = 0.29, *p* = 0.001). The SFS scale for “feeding problems” correlated with PSI “parental distress” (*r* = 0.24, *p* = 0.007) and PSI “dysfunctional parent–child interaction” (*r* = 0.28, *p* = 0.002). “Parental distress” correlated with “dysfunctional parent–child interaction” (*r* = 0.53, *p* = 0.000).

**Table 4:** Bivariate correlation coefficients (according to Pearson) for regulation problems (T1, T2), children`s developmental levels (T1, T2, T3), maternal stress levels and dysfunctionality of the mother-child interaction (T1)

	**SFS C/S T1**	**SFS F T1**	**ASQ C T1**	**ASQ GM T1**	**ASQ FM T1**	**ASQ PS T1**	**ASQ SD T1**	**PSI PD T1**	**PSI DI T1**	**SFS C/S T2**	**SFS F T2**	**ASQ C T2**	**ASQ GM T2**	**ASQ FM T2**	**ASQ PS T2**	**ASQ SD T2**	**ASQ C T3**	**ASQ GM T3**	**ASQ FM T3**	**ASQ PS T3**	**ASQ SD T3**
**SFS C/S T1**	1																				
	*N* = 153																				
**SFS F T1**	.298***	1																			
	*N* = 153	*N* = 153																			
**ASQ C T1**	n.s.	n.s.	1																		
			*N* = 153																		
**ASQ GM T1**	n.s.	n.s.	.393***	1																	
			*N* = 153	N = 153																	
**ASQ FM T1**	n.s.	n.s.	.296***	.376***	1																
			*N* = 153	N = 153	N = 153																
**ASQ PS T1**	n.s.	n.s.	.334***	.434***	.548***	1															
			*N* = 151	N = 151	N = 151	N = 151															
**ASQ SD T1**	n.s.	n.s.	.385***	.389***	.400***	.544***	1														
			N = 152	N = 152	N = 152	N = 150	N = 152														
**PSI PD T1**	.353***	.237**	n.s.	n.s.	n.s.	n.s.	n.s.	1													
	*N* = 127	*N* = 127						N = 127													
**PSI DI T1**	.291***	.277**	n.s.	n.s.	n.s.	n.s.	n.s.	.533***	1												
	*N* = 126	*N* = 126						N = 126	N = 126												
**SFS C/S T2**	.570***	.284**	n.s.	n.s.	n.s.	n.s.	n.s.	.349***	.278**	1											
	N = 140	N = 140						N = 121	N = 120	N = 140											
**SFS F T2**	.223**	.573***	n.s.	n.s.	n.s.	n.s.	n.s.	.305***	.313***	.419***	1										
	N = 140	N = 140						N = 121	N = 120	N = 140	N = 140										
**ASQ C T2**	n.s.	n.s.	.297***	.242**	.235**	.247**	.382***	n.s.	n.s.	n.s	n.s	1									
			N = 137	N = 137	N = 137	N = 135	N = 136					N = 137									
**ASQ GM T2**	n.s.	n.s.	.242**	.292***	.372***	.286***	.171*	n.s.	n.s.	n.s	n.s	.259**	1								
			N = 138	N = 138	N = 138	N = 136	N = 137					N = 137	N = 138								
**ASQ FM T2**	n.s.	n.s.	.211*	.187*	.320***	.252**	.266**	n.s.	n.s.	n.s.	n.s.	.378***	.191*	1							
			N = 134	N = 134	N = 134	N = 132	N = 133					N = 133	N = 134	N = 134							
**ASQ PS T2**	-.173*	n.s.	.221*	.321***	.403***	.296***	.282**	n.s.	n.s.	n.s.	n.s.	.338***	.446***	.292***	1						
	N = 137		N = 137	N = 137	N = 137	N = 135	N = 136					N = 136	N = 137	N = 134	N = 137						
**ASQ SD T2**	n.s.	n.s.	n.s.	.332***	.397***	.401***	.292***	n.s.	n.s.	n.s.	n.s.	.236**	.475***	.247**	.331***	1					
				N = 137	N = 137	N = 135	N = 136					N = 136	N = 137	N = 134	N = 137	N = 137					
**ASQ C T3**	n.s.	n.s.	.311***	.189*	.249**	.208*	.289***	-.228*	-.216*	n.s.	n.s.	.400**	.342***	.339***	.341***	.257**	1				
			N = 123	N = 123	N = 123	N = 121	N = 122	N = 109	N = 108			N = 119	N = 120	N = 116	N = 119	N = 119	N = 123				
**ASQ GM T3**	n.s.	n.s.	n.s.	n.s.	.291***	.353***	.310***	n.s.	n.s.	n.s.	n.s.	.299***	.432***	n.s	n.s.	.449***	.212*	1			
					N = 122	N = 120	N = 121					N = 118	N = 119			N = 118	N = 122	N = 122			
**ASQ FM T3**	n.s.	n.s.	n.s.	.355***	.293***	.297***	.277**	n.s.	n.s.	n.s.	n.s.	.257**	.345***	.281**	.289**	.380***	.344***	.259**	1		
				N = 122	N = 122	N = 120	N = 121					N = 118	N = 119	N = 115	N = 118	N = 118	N = 122	N = 121	N = 122		
**ASQ PS T3**	n.s.	n.s.	n.s.	n.s.	n.s.	n.s.	n.s.	n.s.	n.s.	n.s.	n.s.	.206*	n.s.	n.s	.193*	n.s.	.277**	.193*	.257**	1	
												N = 116			N = 116		N = 120	N = 119	N = 120	N = 120	
**ASQ SD T3**	-.217*	n.s.	.235**	.314***	.446***	.300***	.284***	n.s.	n.s.	-.237**	-.216*	.309***	.459***	.347***	.487***	.487***	.496***	.421***	.509***	.273**	
	N = 123		N = 123	N = 123	N = 123	N = 121	N = 122			N = 122	N = 122	N = 119	N = 120	N = 116	N = 119	N = 119	N = 123	N = 122	N = 122	N = 120	

SFS: Questionnaires on Crying, Feeding and Sleep; C/S: “Crying / Sleep”; F: “Feeding”; PSI: Parental Stress Index; PD: “Parental distress”; DI: “Dysfunctional parent–child interaction”; ASQ: Ages and Stages Questionnaire; C: “Communication”; GM: “Gross motor skills”; FM: “Fine motor skills”; PS: “Problem-solving skills”; SD: “Social development”; ***: *p* ≤ 0.001; **: *p* ≤ 0.01; *p ≤ 0.05; n.s.: not significant.

### Relationships between maternal stress levels and degree of dysfunctionality of mother-child interaction at T1 and T3 and children’s developmental levels at T3

The ASQ scale “social development” at T3 correlated negatively with both PSI scales at T3: “parental distress” (*r* = -0.21, *p* = 0.026) and “dysfunctional parent–child interaction” (*r* = -0.22, *p* = 0.024). Correlations with PSI scales at T1 were not significant.

The ASQ scale “gross motor” at T3 correlated negatively with PSI scale “dysfunctional parent–child interaction” at T3, *r* = -0.20, *p* = 0.044).

Other associations were not significant.

### Bivariate associations between regulatory problems at T1/T2 and the child’s social development at T3

Table 4 shows significant negative correlations between the SFS scale “crying/sleep” at T1 and the ASQ scale “social development” at T3 (*r* = -0.22, *p* = 0.016), whereas the correlation with the SFS scale “feeding problems” at T1 was not significant.

Both SFS-scales correlated negatively at T2 with the ASQ scale “social development” at T3 (for “crying/sleep” *r* = -0.24, *p* = 0.009; for “feeding problems” *r* = -0.22, *p* = 0.017).

### Gender-related differences in regulatory problems and developmental level

No differences were found between the genders in the regulatory problems (SFS) at T1 and T2 (U-tests were not significant). Girls achieved higher values at T3 on the ASQ scales for “fine motor skills” (middle range 70.0 for girls vs. 54.7 for boys, *p* = 0.01) and “social development” (middle range 69.5 for girls vs. 56.1 for boys, *p* = 0.04). *T-*test showed similar results: For “fine motor skills” *M* = 52.32 (*SD* = 8.78) for girls vs. 47.65 (*SD* = 11.31) for boys (*T* = -2.49*, p* = 0.014) and for “social development” *M* = 46.85 (*SD* = 12.30) for girls vs. 41.59 (*SD* = 13.91) for boys (*T* = -2.19*, p* = 0.031).

### Stability of regulatory problems from T1 to T2

The SFS scales showed a highly significant correlation at the first and second measurements. For “SFS Crying, whining and sleep problems,” the correlation coefficient amounted to *r* = 0.57 (*p* = 0.000), and for “SFS Feeding problems,” *r* = 0.57 (*p* = 0.000).

### Prediction of the child’s social development at T3 by means of regulatory problems at T1

The final model proved significant in regression analysis, explaining 13% of the variance in the child’s social development at the third measurement (*R*^*2*^ = 0.21; corrected *R*^*2*^ = 0.13; *F* = 2.57; *p* = 0.011). The child’s social development at T1 proved to be a highly significant predictor (*Beta* = 0.33, *p* = 0.001). The SFS scale for “crying, whining and sleep problems” at T1 was a significant negative predictor (*Beta* = -0.29, *p* = 0.016). The inclusion of control variables in the second model, particularly the variable “social development of the child at T1”, improved the explanatory power of the model significantly. Other variables were not significant. Both SFS scales tested in the first model explained 5% of the variance in social development, with only the scale for “crying, whining and sleep problems” showing a trend towards significance *(Beta* = -0.20, *p* = 0.063) (see Table 5).

**Table 5:** Linear regression analysis (method enter) for investigating the influencing variables at T1 on social development of the child at T3 (N = 97)

**Model summary**	***R***^***2***^	**Corrected *R***^***2***^	***F***	***Beta***	***R***^***2 ***^**Change**
***Model 1***	0.07	0.05	3.45*		
Constant				***	
SFS C/S T1				-0.20	
SFS F T1				-0.13	
***Model 2***	0.21	0.13	2.57*		0.14*
Constant				**	
SFS C/S T1				-0.29*	
SFS F T1				-0.14	
ASQ SD T1				0.33**	
Gender child				0.06	
Gender child x SFS C/S				0.19	
Gender child x SFS F				0.07	
Premature infant				0.01	
PSI PD T1				-0.04	
PSI DI T1				-0.03	
Mother's education				-0.11	
Income/household				0.01	

SFS: Questionnaires on Crying, Feeding and Sleep; C/S: “Crying/Sleep”; F: “Feeding”; PSI: Parental Stress Index; PD : “Parental distress”; DI: “Dysfunctional parent–child interaction”; ASQ: Ages and Stages Questionnaire; SD: “Social development”; ***: *p* ≤ 0.001; **: *p* ≤ 0.01; *p ≤ 0.05; n.s.: not significant.

### Prediction of the child’s social development at T3 by means of regulatory problems at T2

Regression analysis showed a highly significant final model explaining 38% of the variance in social development at the third measurement (*R*^*2*^ = 0.42, corrected *R*^*2*^ = 0.38, *F* = 9.06, *p* = 0.000). The child’s social development at T2 proved to be a highly significant predictor (*Beta* = 0.56, *p* = 0.000), whose inclusion in the regression equation significantly increased the model's explanatory power. The SFS scale for “crying, whining and sleep problems” at T2 proved to be a significant negative predictor (*Beta* = -0.29, *p* = 0.006). Other variables were not significant.

The regulatory problems at T2 alone explained in the first step 8% of the variance (*R*^*2*^ = 0.10, corrected *R*^*2*^ = 0.08, *F* = 5.27, *p* = 0.007). Only the scale for “crying, whining and sleep problems” proved to be significant (*Beta* = -0.27, *p* = 0.016) (see Table 6).

**Table 6:** Linear regression analysis (method enter) for investigating the influencing variables at T2 on the social development of the child at T3 (N = 94)

**Model summary**	***R***^***2***^	**Corrected *R***^***2***^	***F***	***Beta***	***R***^***2 ***^**Change**
***Model 1***	0.10	0.08	5.27**		
Constant				***	
SFS C/S T2				-0.27*	
SFS F T2				-0.10	
***Model 2***	0.42	0.38	9.06***		0.32***
Constant				***	
SFS C/S T2				-0.29**	
SFS F T2				-0.15	
ASQ SD T2				0.56***	
Gender child				0.02	
Gender child x SFS C/S				0.06	
Gender child x SFS F				-0.01	
Premature infant				0.05	
Income/household				0.03	
Mother's education				-0.12	

SFS: Questionnaires on Crying, Feeding and Sleep; C/S: “Crying / Sleep”; F: “Feeding”; ASQ: Ages and Stages Questionnaire; SD: “Social development”; ***: *p* ≤ 0.001; **: *p* ≤ 0.01; *: *p* ≤ 0.05; n.s.: not significant.

### Prediction of other aspects of the child’s development at time 3

The models for the ASQ scales for “communication”, “gross motor skills”, “fine motor skills” and “problem-solving competence” were not significant (see Additional file 1: Tables S7, S8, S9, S10, S11, S12, S13, and S14).

## Discussion

### Influence of regulatory problems on childhood development

The aim of this study was to examine the extent to which regulatory disorders in infants at 4 and 6 months affect childhood developmental levels at 12 months in a high-risk sample. Our results show a statistically significant inverse association between crying, whining and sleep problems at both 4 and 6 months and social development at one year, also after controlling prematurity, developmental level at 4 and 6 months, net income per household and the mother's educational level. The last two sociodemographic control variables did not contribute to explaining the child’s developmental level. This is possibly due to the fact that the high-risk sample belongs to a rather low socioeconomic class, which limited the variance. In conformity with other findings [14], the association between regulatory problems and the children’s social development was relatively weak: In 12-month-old children, approximately 8% of the variance in social development was explained by crying and sleep problems in the 6th month, and the association was even weaker (5% of the variance) in the 4th month, indicating that children’s development is influenced by a range of other factors. In our model, the initial level of development was the strongest predictor, suggesting a continuity of developmental progress. The greater predictive power of the crying and sleep problems in the 6th month, compared to the predictive power of the data recorded in the 4th month, is explained by the shorter period leading up to the time of measurement of the criterion variable (12th month).

Furthermore, it is probable that the focus of problems within the scale for “crying, whining and sleep problems” will shift between the first and second measurements (time period of around 2 months). Since the scale is used to record early regulatory problems and does not allow for a separate view of sleep and crying problems, it is only theoretically possible to gauge whether it is the sleep problems or rather the crying that has influenced the child’s social development. Excessive crying, which is typical of the first months of life, is generally replaced during the course of development by sleep problems [8]. Sleep problems are pushed to the foreground in the 6th and 7th months, accompanied by waking and crying at night and whining during the day caused by the lack of sleep at night and overtiredness.

Persistent crying and sleep problems in early infancy affect both the well-being of parents and the relationship between parents and infant [9]. Thus persistent sleep deprivation leads to a combination of exhaustion, anger and frustration, which can, in turn, lead to feelings of guilt and distinct ambivalence towards the child [19], adversely affecting, in the long term, the relationship with the child and its social development. Under these circumstances, the ability of an exhausted and irritable mother to provide her child with a secure foundation for “social referencing” [29] is limited. Likewise, sleep-deprived, whining infants are less likely to be concerned with exploring the (social) world.

It remains unclear, however, why feeding problems, which are known to be related to an insecure mother-child interaction, had no influence on children’s social development in our study. We found bivariate association between feeding problems at T2 and social development at T3, but the scale “feeding problems” was redundant as a predictor (probably due multicollinearity with the scale “crying/sleep”). Feeding problems between the 4th and 6th months are fairly common and are most frequently observed with the introduction of solid food [6]. Since we have no standard or cut-off values available in the SFS for the diagnosis of feeding disorders, it is difficult to assess whether the feeding problems in this study were significant for a clinical diagnosis. Feeding problems are most likely to be a temporary problem. The gradual process of weaning and the change to solid food present a new developmental task [30], which, like any new developmental task, can lead to (temporary) insecurity on the part of the mother, but does not generally result in serious exhaustion. Clinical observations prove that with minor feeding problems, dysfunction of the relationship is frequently limited only to the feeding situation and interaction at other times seems to remain intact (personal disclosure by K. Scholtes, parent-infant consultation hours, University Hospital of Heidelberg). As mentioned above, the data pertaining to maternal distress levels for the second measurement period are not available to us, making it impossible to empirically support these hypotheses.

A key question requiring consideration is why crying and sleep problems in our study exerted influence on only one aspect of development, i.e. social development. Using the Griffiths scales in their assessment, Wolke and colleagues [14] observed that the regulatory problems of 5-month-old infants prescribed a lower level of development in general in the same infants at 20 months. In our study, we separately tested the five aspects of development: gross motor skills, fine motor skills, communication, problem-solving competence and social development. Our assumption is that social development is influenced by the parent–child relationship to a greater extent than, for example, motor skill development. This could explain why it is more vulnerable to the stress caused by regulatory problems. Furthermore, it is likely that mothers' response behaviour in the questionnaire with respect to social development was influenced more by their mental state than when responding to questions regarding, for instance, their children’s motor skill development. In the questionnaire, items concerning motor skill development can also be tested more directly than items pertaining to social development. Thus it is possible that mothers who are more exposed to stress and are possibly depressed, judge their child’s social development more negatively [20].

In our study, regulatory problems had no influence on cognitive aspects of development, communication (forerunners of language) and problem-solving competence. It is possible that the time of measurement at around one year was too early to discover the influence of regulatory problems in infancy on cognitive development. With respect to the long-term consequences of early regulatory disorders in cognitive development, the available evidence is limited. It is also not clear whether early regulatory disorders have anything to do with temporary delays in development [12,14]. Wolke and colleagues (ibid.) found a very small but significant influence of regulatory problems on cognitive development (measured at 56 months). Rao and colleagues (ibid.) also reported an association between regulatory problems and cognitive development in infants at 60 months. It is conceivable that any detailed and reliable recording of cognitive development at 12 months may be problematic.

### Link between maternal distress during the children’s 4th month and their subsequent social development at 12 months

Our next hypothesis, which proclaimed an association between parental distress in the infant's 4th month and their subsequent social development at 12 months, could not be confirmed. Neither maternal distress nor the dysfunctionality of interaction at the age of 4 months alone had the predictive power to explain the variance in children’s social development at 12 months. Thus we could not replicate the observation of Salomonsson and Sleed [20]. However, we found negative correlations between maternal distress (*r* = -0.21), dysfunctionality of interaction (*r* = -0.22) and the children’s social development at the age of 12 months when they were concurrently measured. Beyond that, we found an inverse correlation between “gross motor” scale at 12 months and “dysfunctional parent–child interaction” at T3 (*r* = -0.20). The direction of this association, however, remains unclear, although it may be pertinent to ask whether impairment of the mother's well-being, which frequently but not automatically places a strain on the mother-child relationship, could, indirectly, have a negative effect on the child’s motor development.

### Link between regulatory disorders, maternal stress levels and degree of dysfunctionality of mother-child interaction during the children’s 4^th^ month

In this study, crying and sleeping problems in 4-month-old infants are associated with increased maternal stress levels (*r* = 0.35) and dysfunctionality of mother-child interaction reported by mothers (*r* = 0.29) at the same measurement point. Furthermore, we found a strong association between maternal stress levels and the degree of dysfunctionality of mother-child interaction (*r* = 0.53). These results emphasize the transactional nature of the “triad of symptoms” [3,9] between the infant's problems with behavior regulation, the occurrence of dysfunctional interaction patterns between the infant and care-giver, and parents’ stress level. As at-risk families have access to relatively few resources, the relationship between parents and infant can be seriously influenced by problems such as early regulation disorders coupled with psychosocial pressures [3,9].

The data for maternal distress were not collected during measurement at 6 months but a similar result can be assumed.

### Gender differences

Our study did not reveal any moderating effect of gender on the association between regulatory problems and developmental level, unlike the study by Wolke and colleagues [14], which had found a more pronounced association among boys. We were also unable to find any gender differences relating to regulatory problems. The paucity of available data on gender differences with respect to early regulatory problems suggests, on the whole, no (or only slight) differences [31]. That being said, some research teams (e.g. [13]) have found significantly higher rates for boys compared to girls.

One-year-old girls reached a higher level of development in areas of social development and fine motor skills. Our results support observations suggesting more advanced development in girls [32].

### Limitations

The ASQ, used to record children’s developmental levels based on mothers' assessments, does not provide objective data, as mothers' assessments can be influenced by their well-being and can therefore lead to the anomalies mentioned above. In addition, the ASQ was designed as a screening tool to measure delays in development. Therefore, the upper range of development is not represented in enough detail (ceiling effect), limiting the mappable variance in developmental levels. These limitations of the ASQ demand caution in the interpretation of results.

It is not clear why in both regression models the influence of the crying/sleeping scale on social development increases slightly after adding the covariate/control variables. Perhaps there are other confounding factors, as adding of the scale “social development” (T1, T2), influencing the association between these variables.

Furthermore, as we did not collect data on PSI scales at Time 2, it was not possible to use the same set of predictors for Time 1 and Time 2 regression models.

Having applied regression as the calculation method, we are aware of the fact that it precludes assumptions about causal associations between early regulatory problems and developmental levels. Another limitation of our study is that some of the associations described were cross-sectional (concurrent). Therefore, the directions of the effects between these factors could not be determined.

It should also be noted that our study deals with an at-risk sample, limiting the generalizability of results with respect to other samples. It can also be assumed that the test participants, who were exposed to psychosocial stress, had several problems while filling out the questionnaires, which could have contributed to possible distortions in the response behavior.

### Further research

It would be important to replicate our results in a study based on methods that allow more precise measurements of early regulatory problems such as crying and sleeping. This would also enable separate assessments of crying and sleeping problems. A further suggestion would be to control infants' medical conditions for a better understanding of their impact on regulatory problems. Because no valid data were collected on the infants` medical conditions (i.e. gastrointestinal reflux), it was not possible in this study to determine how infants' physical pain might have influenced their regulatory problems. In future studies, this potential medical impact on children’s development should be included as a covariate.

It would be important, also, to test in the pathway model the role of maternal distress as a mediator between sleeping and crying problems and the child`s social development.

Due to the possible influence of mother's chronic sleep deprivation on the mother-child interaction and therefore on child`s social development, it would be interesting to gather sleep diary data separately from mothers as well.

## Conclusions

Our results reinforce existing knowledge pertaining to the transactional relationship between regulatory problems in infants, maternal distress and dysfunctionality of mother-child interactions. They also provide evidence of a slight but distinct negative influence of crying and sleeping problems on children’s social development. Mothers at risk are likely to be more challenged by difficulties with their children and have fewer resources, such as social support or access to counseling services, than their more fortunate counterparts [23]. This in turn may contribute to the broadening of the children’s initial regulatory problems into other areas of behavior.

As a result, the healthcare system, particularly doctors and midwives, and also family members, should be made more aware of the vulnerabilities of young mothers and more support should be provided to families. In the case of severe regulatory problems, it is advisable to draw parents’ attention to the parent-infant advisory services, which not only help improve early childhood regulatory problems but also support mother-child interaction and help relieve pressure on young families.

Easy access support services provided by e.g. family health visitors, particularly to the so-called “high-risk families”, are also recommended. Services offering early assistance after childbirth, especially home-visiting programs (e.g. the KfdN prevention project, or comparable projects), which have proved effective in relation to children’s social development and reducing dysfunctionality of mother-child interaction [33], can be a sensible addition in the outreach context.

## Endnote

^a^The project “Nobody slips through the net” is a psychosocial primary and secondary prevention program for families with children in their first year of life in a total of 11 districts in the states Hessen, Baden-Württemberg and the whole state of Saarland. The key components consist of a course for parents, family home visits mainly through health visitors and the initiation of a local network with a coordination point for the support organisation [34].

## Competing interests

The authors declare that they have no competing interests.

## Authors’ contributions

AS conducted and coordinated the study, performed the statistical analysis and drafted the manuscript. CF conducted the study and contributed critical comments on the manuscript. AE coordinated the project with KfdN and contributed critical comments on the manuscript. MC conceived of the study and contributed critical comments on the manuscript. All authors read and approved the final manuscript.

## Supplementary Material

Additional file 1: Table S7 Linear regression analysis (method enter) for investigating the influencing variables at T1 on gross motor skills of the child at T3 (N = 98). **Table S8.** Linear regression analysis (method enter) for investigating the influencing variables at T2 on gross motor skills of the child at T3 (N = 96). **Table S9.** Linear regression analysis (method enter) for investigating the influencing variables at T1 on fine motor skills of the child at T3 (N = 97). **Table S10.** Linear regression analysis (method enter) for investigating the influencing variables at T2 on fine motor skills of the child at T3 (N = 96). **Table S11.** Linear regression analysis (method enter) for investigating the influencing variables at T1 on problem solving skills of the child at T3 (N = 99). **Table S12.** Linear regression analysis (method enter) for investigating the influencing variables at T2 on problem solving skills of the child at T3 (N = 97). **Table S13.** Linear regression analysis (method enter) for investigating the influencing variables at T1 on communication development of the child at T3 (N = 98). **Table S14.** Linear regression analysis (method enter) for investigating the influencing variables at T2 on communication development of the child at T3 (N = 97). Click here for file
