# Supplementary material for: Influence of early regulatory problems in infants on their development at 12 months: a longitudinal study in a high-risk sample
Source: Child Adolesc Psychiatry Ment Health. 2013 Oct 12;7:35. doi: 10.1186/1753-2000-7-35 (PMC3854693; doi:10.1186/1753-2000-7-35)
Supplement: Additional file 1: Table S7 — Linear regression analysis (method enter) for investigating the influencing variables at T1 on gross motor skills of the child at T3 (N = 98). Table S8. Linear regression analysis (method enter) for investigating the influencing variables at T2 on gross motor skills of the child at T3 (N = 96). Table S9. Linear regression analysis (method enter) for investigating the influencing variables at T1 on fine motor skills of the child at T3 (N = 97). Table S10. Linear regression analysis (method enter) for investigating the influencing variables at T2 on fine motor skills of the child at T3 (N = 96). Table S11. Linear regression analysis (method enter) for investigating the influencing variables at T1 on problem solving skills of the child at T3 (N = 99). Table S12. Linear regression analysis (method enter) for investigating the influencing variables at T2 on problem solving skills of the child at T3 (N = 97). Table S13. Linear regression analysis (method enter) for investigating the influencing variables at T1 on communication development of the child at T3 (N = 98). Table S14. Linear regression analysis (method enter) for investigating the influencing variables at T2 on communication development of the child at T3 (N = 97). [file 1753-2000-7-35-S1.doc]

Additional file 1

**Table S7**: Linear regression analysis (method enter) for investigating the influencing variables at T1 on gross motor skills of the child at T3 (N = 98)

| **Model summary** | ***R^2^*** | **Corrected *R^2^*** | ***F*** | ***Beta*** | ***R^2^* Change** |
| --- | --- | --- | --- | --- | --- |
| ***Model 1*** | 0.01 | -0.01 | 0.66 (n.s.) |  |  |
| Constant |  |  |  | *** |  |
| SFS C/S T1 |  |  |  | n.s |  |
| SFS F T1 |  |  |  | n.s. |  |
| ***Model 2*** | 0.05 | -0.03 | 0.66 (n.s.) |  | n.s. |
| Constant |  |  |  | n.s. |  |
| SFS C/S T1 |  |  |  | n.s |  |
| SFS F T1 |  |  |  | n.s |  |
| ASQ GM T1 |  |  |  | n.s |  |
| Gender child |  |  |  | n.s |  |
| Gender child x SFS C/S |  |  |  | n.s. |  |
| Gender child x SFS  F |  |  |  | n.s |  |
| Premature infant |  |  |  | n.s |  |
| PSI PD T1 |  |  |  | n.s |  |
| PSI DI T1 |  |  |  | n.s |  |
| Mother's education |  |  |  | n.s |  |
| Income / household |  |  |  | n.s |  |

SFS: Questionnaires on Crying, Feeding and Sleep; C/S: “Crying / Sleep”; F: “Feeding”; PSI: Parental Stress Index; PD : “Parental distress“; DI: "Dysfunctional parent-child interaction; ASQ: Ages and Stages Questionnaire; GM: “Gross motor skills” ; ***: *p* ≤ 0.001; n.s.: not significant

**Table S8:** Linear regression analysis (method enter) for investigating the influencing variables at T2 on gross motor skills of the child at T3 (N = 96)

| **Model summary** | ***R^2^*** | **Corrected *R^2^*** | ***F*** | ***Beta*** | ***R^2^* Change** |
| --- | --- | --- | --- | --- | --- |
| ***Model 1*** | 0.004 | -0.01 | 0.25 (n.s.) |  |  |
| Constant |  |  |  | *** |  |
| SFS C/S T1 |  |  |  | n.s |  |
| SFS F T1 |  |  |  | n.s. |  |
| ***Model 2*** | .20 | .14 | 3.76*** |  | 0.19*** |
| Constant |  |  |  | n.s. |  |
| SFS C/S T1 |  |  |  | n.s |  |
| SFS F T1 |  |  |  | n.s |  |
| ASQ GM T2 |  |  |  | .41*** |  |
| Gender child |  |  |  | n.s |  |
| Gender child x SFS C/S |  |  |  | n.s. |  |
| Gender child x SFS  F |  |  |  | n.s |  |
| Premature infant |  |  |  | n.s |  |
| Mother’s education |  |  |  | n.s |  |
| Income / household |  |  |  | n.s |  |

SFS: Questionnaires on Crying, Feeding and Sleep; C/S: “Crying/ Sleep”; F: “Feeding”; ASQ: Ages and Stages Questionnaire; GM: „Gross motor skills; ***: *p* ≤ 0.001; n.s.: not significant

**Table S9**: Linear regression analysis (method enter) for investigating the influencing variables at T1 on fine motor skills of the child at T3 (N = 97)

| **Model summary** | ***R^2^*** | **Corrected *R^2^*** | ***F*** | ***Beta*** | ***R^2^* Change** |
| --- | --- | --- | --- | --- | --- |
| ***Model 1*** | 0.03 | 0.01 | 1.60 (n.s.) |  |  |
| Constant |  |  |  | *** |  |
| SFS C/S T1 |  |  |  | n.s. |  |
| SFS F T1 |  |  |  | n.s. |  |
| ***Model 2*** | 0.17 | 0.10 | 2.43* |  | 0.14* |
| Constant |  |  |  | * |  |
| SFS C/S T1 |  |  |  | n.s. |  |
| SFS F T1 |  |  |  | n.s. |  |
| ASQ FM T1 |  |  |  | 0.29** |  |
| Gender child |  |  |  | n.s. |  |
| Gender child x SFS C/S |  |  |  | n.s. |  |
| Gender child x SFS  F |  |  |  | n.s. |  |
| Premature infant |  |  |  | n.s. |  |
| PSI PD T1 |  |  |  | n.s. |  |
| PSI DI T1 |  |  |  | n.s. |  |
| Mother’s education |  |  |  | n.s. |  |
| Income / household |  |  |  | n.s. |  |

SFS: Questionnaires on Crying, Feeding and Sleep; C/S: “Crying/ Sleep”; F: “Feeding”; PSI: Parental Stress Index; PD : “Parental distress“; DI: “Dysfunctional parent-child interaction; ASQ: Ages and Stages Questionnaire; FM: „Fine motor skills“; ***: *p* ≤ 0.001; **: *p* ≤ 0.01; **p* ≤ 0.05; n.s.: not significant

**Table S10:** Linear regression analysis (method enter) for investigating the influencing variables at T2 on fine motor skills of the child at T3 (N = 96)

| **Model summary** | ***R^2^*** | **Corrected *R^2^*** | ***F*** | ***Beta*** | ***R^2^* Change** |
| --- | --- | --- | --- | --- | --- |
| ***Model 1*** | 0.001 | -0.02 | 0.06 (n.s.) |  |  |
| Constant |  |  |  | *** |  |
| SFS C/S T1 |  |  |  | n.s |  |
| SFS F T1 |  |  |  | n.s. |  |
| ***Model 2*** | 0.14 | 0.08 | 2.34* |  | 0.14** |
| Constant |  |  |  | n.s. |  |
| SFS C/S T1 |  |  |  | n.s |  |
| SFS F T1 |  |  |  | n.s |  |
| ASQ FM T2 |  |  |  | 0.29** |  |
| Gender child |  |  |  | n.s |  |
| Gender child x SFS C/S |  |  |  | n.s. |  |
| Gender child x SFS  F |  |  |  | n.s |  |
| Premature infant |  |  |  | n.s |  |
| Mother's education |  |  |  | n.s |  |
| Income / household |  |  |  | n.s |  |

SFS: Questionnaires on Crying, Feeding and Sleep; C/S: “Crying/ Sleep”; F: “Feeding”; ASQ: Ages and Stages Questionnaire; FM: “Fine motor skills”; ***: *p* ≤ 0.001; **: *p* ≤ 0.01; **p* ≤ 0.05; n.s.: not significant

**Table S11**: Linear regression analysis (method enter) for investigating the influencing variables at T1 on problem solving skills of the child at T3 (N = 99)

| **Model summary** | ***R^2^*** | **Corrected *R^2^*** | ***F*** | ***Beta*** | ***R^2^* Change** |
| --- | --- | --- | --- | --- | --- |
| ***Model 1*** | 0.00 | -0.02 | 0.03 (n.s.) |  |  |
| Constant |  |  |  | *** |  |
| SFS C/S T1 |  |  |  | n.s. |  |
| SFS F T1 |  |  |  | n.s. |  |
| ***Model 2*** | 0.16 | 0.09 | 2.19* |  | 0.16* |
| Constant |  |  |  | * |  |
| SFS C/S T1 |  |  |  | n.s. |  |
| SFS F T1 |  |  |  | n.s. |  |
| ASQ PS T1 |  |  |  | n.s. |  |
| Gender child |  |  |  | n.s. |  |
| Gender child x SFS C/S |  |  |  | 0.41* |  |
| Gender child x SFS  F |  |  |  | n.s. |  |
| Premature infant |  |  |  | n.s. |  |
| PSI PD T1 |  |  |  | n.s. |  |
| PSI DI T1 |  |  |  | -0.36*** |  |
| Mother's education |  |  |  | n.s. |  |
| Income / household |  |  |  | n.s. |  |

SFS: Questionnaires on Crying, Feeding and Sleep; C/S: “Crying/ Sleep”; F: “Feeding”; PSI: Parental Stress Index; PD : “Parental distress“; DI: "Dysfunctional parent-child interaction“; ASQ: Ages and Stages Questionnaire; ;PS: “Problem solving“; ***: *p* ≤ 0.001; **p* ≤ 0.05; n.s.: not significant

**Table S12:** Linear regression analysis (method enter) for investigating the influencing variables at T2 on problem solving skills of the child at T3 (N = 97)

| **Model summary** | ***R^2^*** | **Corrected *R^2^*** | ***F*** | ***Beta*** | ***R^2^* Change** |
| --- | --- | --- | --- | --- | --- |
| ***Model 1*** | 0.03 | 0.01 | 1.49 (n.s.) |  |  |
| Constant |  |  |  | *** |  |
| SFS C/ S T1 |  |  |  | n.s |  |
| SFS F T1 |  |  |  | n.s. |  |
| ***Model 2*** | 0.09 | 0.03 | 1.48 (n.s) |  | n.s. |
| Constant |  |  |  | n.s. |  |
| SFS C/ S T1 |  |  |  | n.s |  |
| SFS F T1 |  |  |  | n.s |  |
| ASQ PS T2 |  |  |  | 0.24* |  |
| Gender child |  |  |  | n.s |  |
| Gender child x SFS C/S |  |  |  | n.s. |  |
| Gender child x SFS F |  |  |  | n.s |  |
| Premature infant |  |  |  | n.s |  |
| Mother's education |  |  |  | n.s |  |
| Income / household |  |  |  | n.s |  |

SFS: Questionnaires on Crying, Feeding and Sleep; C/S: “Crying/ Sleep”; F: “Feeding”; ASQ: Ages and Stages Questionnaire; PS: “Problem solving“; ***: *p* ≤ 0.001; **p* ≤ 0.05; n.s.: not significant

**Table S13**: Linear regression analysis (method enter) for investigating the influencing variables at T1 on communication development of the child at T3 (N = 98)

| **Model summary** | ***R^2^*** | **Corrected *R^2^*** | ***F*** | ***Beta*** | ***R^2^* Change** |
| --- | --- | --- | --- | --- | --- |
| ***Model 1*** | 0.02 | 0.00 | 1.02 (n.s.) |  |  |
| Constant |  |  |  | *** |  |
| SFS C/S T1 |  |  |  | n.s. |  |
| SFS F T1 |  |  |  | n.s. |  |
| ***Model 2*** | 0.19 | 0.12 | 2.76** |  | 0.17** |
| Constant |  |  |  | n.s. |  |
| SFS C/S T1 |  |  |  | n.s. |  |
| SFS F T1 |  |  |  | n.s. |  |
| ASQ C T1 |  |  |  | 0.31*** |  |
| Gender child |  |  |  | n.s. |  |
| Gender child x SFS C/S |  |  |  | n.s. |  |
| Gender child x SFS  F |  |  |  | n.s. |  |
| Premature infant |  |  |  | n.s. |  |
| PSI PD T1 |  |  |  | n.s. |  |
| PSI DI T1 |  |  |  | -0.22* |  |
| Mother's education |  |  |  | n.s. |  |
| Income / household |  |  |  | n.s. |  |

SFS: Questionnaires on Crying, Feeding and Sleep; C/S: “Crying/ Sleep”; F: “Feeding”; PSI: Parental Stress Index; PD : “Parental distress“; DI: "Dysfunctional parent-child interaction“; ASQ: Ages and Stages Questionnaire; ;C: “Communication“; ***: *p* ≤ 0.001; **: *p* ≤ 0.01; **p* ≤ 0.05; n.s.: not significant

**Table S14**: Linear regression analysis (method enter) for investigating the influencing variables at T2 on communication development of the child at T3 (N = 97)

| **Model summary** | ***R^2^*** | **Corrected *R^2^*** | ***F*** | ***Beta*** | ***R^2^* Change** |
| --- | --- | --- | --- | --- | --- |
| ***Model 1*** | 0.03 | 0.01 | 1.54 (n.s.) |  |  |
| Constant |  |  |  | *** |  |
| SFS C/ S T1 |  |  |  | n.s |  |
| SFS F T1 |  |  |  | n.s. |  |
| ***Model 2*** | 0.21 | 0.16 | 4.02 *** |  | 0.18*** |
| Constant |  |  |  | n.s. |  |
| SFS C/ S T1 |  |  |  | n.s |  |
| SFS F T1 |  |  |  | n.s |  |
| ASQ C T2 |  |  |  | 0.42*** |  |
| Gender child |  |  |  | n.s |  |
| Gender child x SFS C/S |  |  |  | n.s. |  |
| Gender child x SFS  F |  |  |  | n.s |  |
| Premature infant |  |  |  | n.s |  |
| Mother's education |  |  |  | n.s |  |
| Income / household |  |  |  | n.s |  |

SFS: Questionnaires on Crying, Feeding and Sleep; C/S: “Crying/ Sleep”; F: “Feeding”; ASQ: Ages and Stages Questionnaire; C: “Communication“; ***: *p* ≤ 0.001; ** n.s.: not significant.
